# Primary anorectal melanoma mimicking polyp in a scleroderma patient: a case report

**DOI:** 10.1097/MS9.0000000000000321

**Published:** 2023-03-27

**Authors:** Osama Alazki, Hasan Othman, Rita Mohammad, Jacob Al-Dabbagh, Lina Al-Soufi, Zuheir Alshehabi, Samir Kanaan

**Affiliations:** a Faculty of Medicine; b Cancer Research Center, Tishreen University; c Department of General Surgery; d Department of Pathology, Tishreen University Hospital; e Department of Dermatology, National Hospital of Latakia, Latakia, Syria

**Keywords:** abdominoperineal resection, case report, malignant melanoma, primary anorectal melanoma, scleroderma

## Abstract

Primary anorectal melanoma is an extremely rare malignancy of the gastrointestinal tract with a very poor prognosis. Due to the late presentation, most patients with primary anorectal melanoma are diagnosed at advanced stages. Scleroderma is an autoimmune disease characterized by fibrosis of the skin and visceral organs. There is an increased risk of developing cancer in scleroderma patients.

**Case Presentation:**

A 57-year-old Syrian female diagnosed with localized scleroderma complained of a sensation of a mass in her anal area. She was diagnosed with primary rectal melanoma and was put on neoadjuvant radiotherapy. Following the radiotherapy, the endoscopy revealed several black lesions in her anal canal, and thus abdominoperineal resection was conducted.

**Discussion and Conclusion:**

Malignant melanoma can occur in unsuspected locations such as the anal canal. Novel therapies like anti-CTLA4 drugs have proven efficient in controlling the disease. The lack of data in the literature on this malignancy and the absence of guidelines make it challenging for an optimal approach.

## Introduction

Highlights

- Malignant melanoma can occur in rare and nonsuspected locations such as the anal canal, which can manifest as a polyp and mimic other pathologies.
- Scleroderma patients are at high risk of developing cancer, especially melanoma and other skin malignancies.
- Although there is no reliable guideline, surgery is the main treatment option for anorectal melanoma, with a preference for radical approaches.
- Immunotherapy (anti-CTLA4 and programmed death-1 inhibitors) has proved effective in treating mucosal melanoma such as nivolumab and in lowering the recurrence rate.
- Immunotherapy can aggravate or increase the risk of scleroderma in melanoma patients.

Primary anorectal melanoma (PARM) is an extremely rare malignancy of the gastrointestinal (GI) tract with a very poor prognosis; it only constitutes 0.05% of colorectal cancers and about 1% of all malignant melanomas^1^. Despite its rarity, it still represents the third most frequent primary melanoma, following cutaneous and ocular melanoma, and its incidence continues to increase^2^. It is slightly more common in females and usually occurs in the fifth or sixth decade of life^1^. On the other hand, localized scleroderma (morphea) is a rare inflammatory cutaneous disorder that varies from an isolated lesion to a severe disseminated pattern^3^,^4^. However, this entity has been linked with an increased risk of developing cancer such as melanoma and other skin malignancies^4^,^5^.

Herein, we present a scleroderma patient who was diagnosed with PARM and the treatment strategies we used to cure her disease. This case has been reported in line with the SCARE (Surgical CAse REport) 2020 criteria^5^.

## Case presentation

A 57-year-old female presented to her gastroenterologist with a 1-week aching, intermittent, mild, nonradiating lower abdominal pain that was getting worse with time and was slightly relieved after defecation, accompanied with a nonpainful and nonprotruding mass sensation in her anal canal, and altered bowel habits. The patient is a nonsmoker, nonalcoholic and has no history of malignancies in her family. She denied hematochezia, weight loss, or pruritus. She has a history of localized scleroderma, which was diagnosed based on the physical examination 10 years ago, and was treated with topical corticosteroids. The skin biopsy procedure was refused by the patient; thus, the diagnosis was not confirmed histopathologically.

The general physical exam reported that the patient looked slightly pale with no palpable masses in her abdomen. Her blood results revealed she was a little anaemic [haemoglobin (Hb)=11.5 g/dl, mean corpuscular haemoglobin (MCH)=25 pg/RBC, red blood cell count (RBC)=4.59×106/mm^3^], her white blood cells count was on the upper higher limit, erythrocyte sedimentation rate was 50 mm/h in the first hour and C-reactive protein was 14.5 mg/l. Initial diagnoses were anorectal carcinoma, a benign polyp and haemorrhoids. However, the clinical and laboratory findings suggested rectal neoplasm.

**Figure 1:**
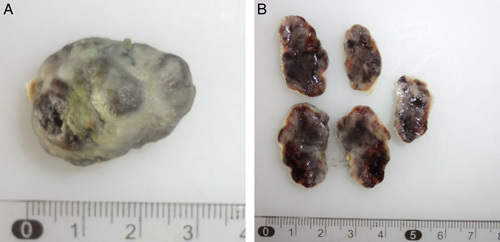
Gross inspection of the excised anorectal polyp: (A) soft polypoid lobulated mass measuring 2.5×1.5 cm and (B) serial sections of the polyp revealing solid cut heamorrhagic surface.

The lower GI endoscopy revealed a pigmented polyp located 3 cm from the anal verge and measuring ∼2.5×1.5 cm in diameter, in addition to external haemorrhoids and skin tags in the anal canal. The polyp was entirely excised by endoscopy and sent for a histological examination (Fig. 1).

**Figure 2:**
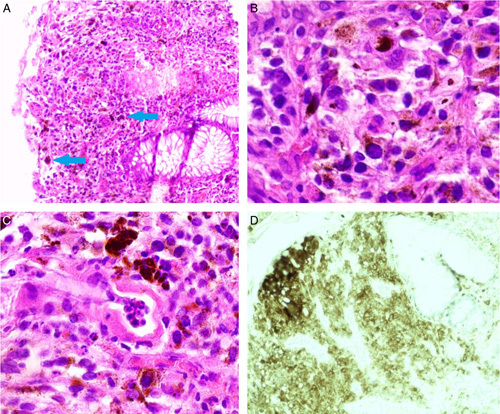
The histopathology examination of the excised polyp revealed: (A) pigmented polygonal and spindle cells infiltrate (arrows) [hematoxylin and eosin (H&E) ×40], (B) melanin deposits and nesting pattern of neoplastic cells (H&E ×100), (C) neoplastic melanocytes with higher magnification (H&E ×200) and (D) immunohistochemistry staining shows positivity for S100.

Histopathological findings of the polypoid tissue were compatible with malignant melanoma, and tumour margins were not identified due to the cauterization effect. Immunohistochemistry staining was positive for S100 (which was the only staining available), confirming the diagnosis of malignant melanoma (Fig. 2). The patient has been tested for *BRAF V600E* gene mutation, which was negative (wild type), excluding her from any cancer-targeted therapy.

**Figure 3:**
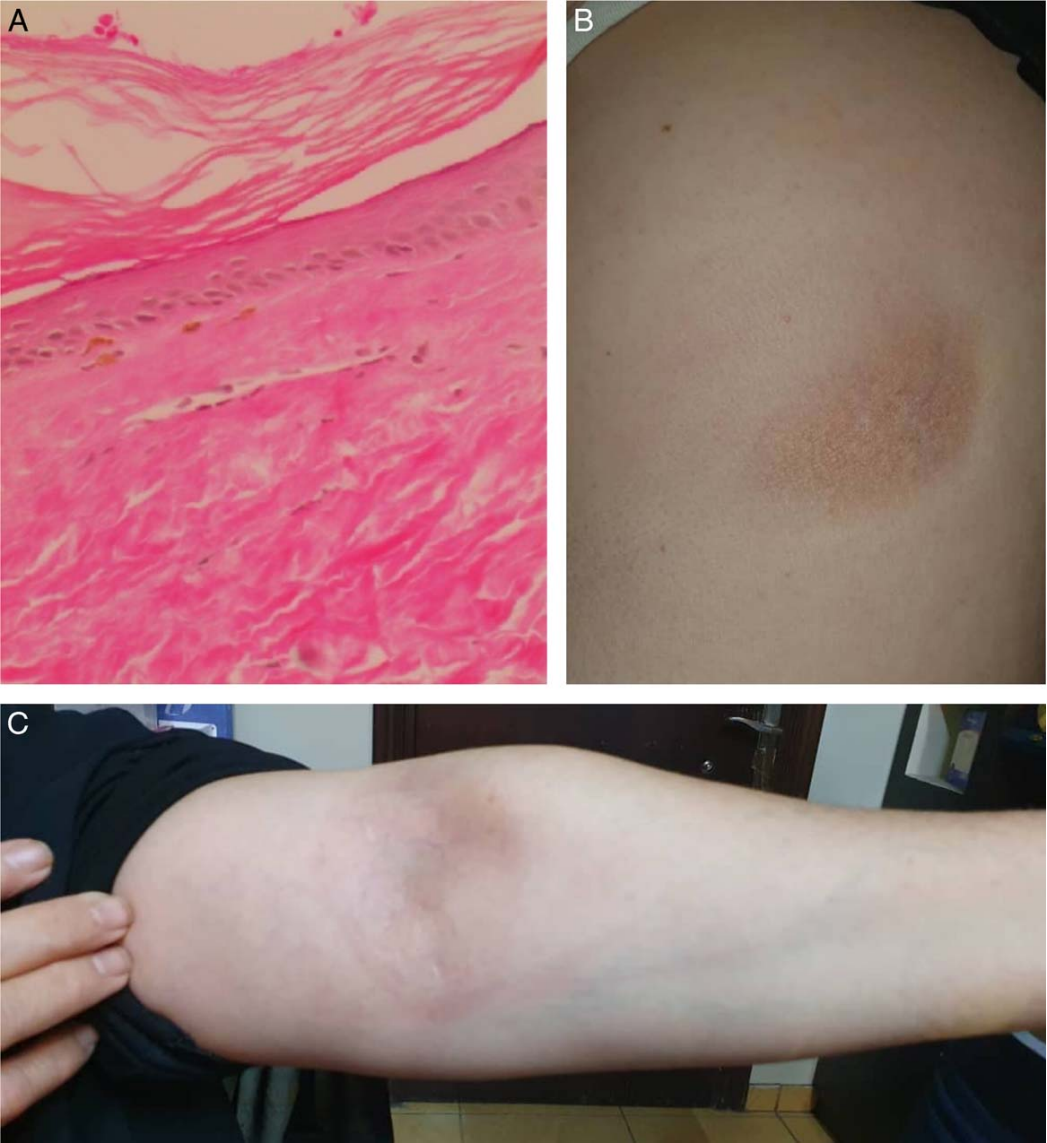
Features of scleroderma: (A) microscopic inspection revealed epidermal atrophy and thickened collagen bundles within the papillary and reticular dermis (hematoxylin and eosin ×200). Dermatological exam after the diagnosis of primary anorectal melanoma: (B) initial morphea lesion on patient’s trunk (before biopsy) and (C) localized lesion on patient’s cubital fossa.

After a short period, her doctors noticed an increase in the size of the initial morphea with the development of new lesions under the breasts, periumbilical and on both cubital fossae (Fig. 3B, C), In addition to a pigmented spot on her right leg. Anyway, she was treated then with methotrexate. A skin biopsy for the initial morphea on her trunk was performed and confirmed the diagnosis of scleroderma (Fig. 3A). A full body contrast computed tomography (CT) scan revealed no abnormalities or any other masses; inguinal and pelvic lymph nodes were also unremarkable.

Neoadjuvant radiotherapy was started in hopes of minimizing the remaining lesions and only conducting a wide local excision rather than radical excision with abdominoperineal resection (APR), avoiding a permanent colostomy and sparing anal sphincters. A month later, she was scheduled for a full body ^18^F-FDG PET/CT (positron emission tomography with 2-deoxy-2-[fluorine-18]fluoro-D-glucose integrated with computed tomography) multislice scan, which revealed normal metabolic activity in the lungs, liver and brain; no visible abnormal fluorodeoxyglucose accumulation in pelvic lymph nodes, confirming PARM.

After her radiotherapy, another lower GI endoscopy revealed blackish-pigmented lesions where the original lesion was. Due to melanoma being a highly aggressive neoplasm, the primary lesion was close to the sphincter, and to prevent recurrences, a decision was made by her oncologist and general surgeon with the patient’s consent to radically excise the rectum and sigmoid colon with APR and to put a permanent colostomy. The surgery was conducted at the university hospital and lasted around 3 h with no major complications during or after the procedure. The patient was discharged with a good general status.

Macroscopic inspection of the dissected rectal revealed several dark spots adjacent to the primary polyp extending 4 cm upward from the dentate line. Microscopic examination revealed metastasis in one of the total excised lymph nodes. To ensure the prevention of any recurrences, the patient was put on 20 doses of nivolumab. During the patient’s last follow-up visit, the CT scan and laboratory tests were normal.

## Discussion

PARM is a rare entity comprising a small percentage of all the cases of melanoma and GI malignancies. However, the anal canal is the most common site for primary mucosal melanoma^1^. This malignancy mostly originates from melanocytes adjacent to the dentate line^6^, an area with rich vascularity which may explain the high tendency of this tumour to give early distant metastases; the liver is the most involved site^7^,^8^. The most common symptoms of PARM are rectal bleeding symptoms, pain, rectal mass and changes in bowel movements^6^. These nonspecific symptoms mimic other anorectal disorders, which can delay the early detection of PARM^9^,^10^. Due to the late presentation, most patients with PARM are diagnosed at advanced stages, and thus they have a very poor prognosis with a 5-year survival rate of 6–22%^8^,^11^. The earlier presentation may have a huge effect in improving the already poor prognosis of the disease^11^.

The aetiology of PARM is not the same as that of cutaneous melanoma and is not yet clearly identified^7^. Ultraviolet radiation – which is the main risk factor for cutaneous melanoma – is not involved in the pathogenesis of PARM^8^. However, a few risk factors have been identified, such as immunosuppressed individuals and those descending from the Caucasian race^7^,^12^; our patient is none of the aforementioned.

Scleroderma is considered a risk factor for cancer, especially skin cancers like melanoma^13^. The mechanism for this risk is still complicated and under study, but it is thought that cytotoxic therapies for scleroderma are associated with an increased risk of cancer^4^,^13^. Nonetheless, our patient was treated only with topical steroids.

PARM lesions lack pigmentation in about one-third of all cases^14^. Therefore, they are mistaken for polyps, rectal cancer, or haemorrhoids^9^,^15^. Moreover, most GI melanomas are metastases of unknown primary cutaneous melanoma^7^.

Immunohistochemistry plays a pivotal role in differentially diagnosing the tumour from other rare pigmented malignancies, which can change the whole treatment plan and surgical procedure. HMB45, Melan-A and S100 are the main immunohistochemistry panels in diagnosing malignant melanoma^6^. S100 was the only staining available, confirming the diagnosis in our case.

Due to the limited number of cases, evidence-based guidelines related to the treatment and follow-up of PARM are not yet available^15^. Thus, most of the treatment strategies for PARM are extrapolated from the guidelines regarding the treatment of cutaneous melanoma^1^. It is agreeable that surgery is the cornerstone therapeutic option for PARM, as the current surgical techniques include either wide local excision which spares the sphincter or the more extensive APR, which provides better local disease control^16^. However, there is no significant difference in overall survival rate and currently no consensus on which one of the two mainstay surgical techniques is preferred^16^,^17^.

Some cases report cancer-free patients at more than 10 months of follow-up with neoadjuvant chemoradiotherapy or radiotherapy despite the role of chemotherapy and radiotherapy being limited since the tumour is usually resistant to these therapies^9^,^18^.

Targeting *BRAF* gene mutation is the main component of the systemic treatment plan for melanoma^19^. Anyway, only 50% of cases with malignant melanoma are positive for *BRAF* gene mutation^19^. However, our patient was negative for any *BRAF* gene mutations.

Considering that melanoma is an immounosensitive malignancy, novel therapies like anti-CTLA4 like ipilimumab and PD-1 inhibitor agents like nivolumab have proven beneficial in the treatment of mucosal melanomas and may provide the same advantages to patients with PARM despite the response rate being lower in mucosal lesions^20^. As with our patient’s current state, the use of nivolumab as a PD-1 inhibitor agent is thought to have a positive effect on lowering the recurrence rate better than anti-CTLA4 agents^20^. However, it is linked with an increased risk of developing or exacerbating scleroderma lesions^21^, which can become a nuisance in treating PARM.

## Conclusion

Malignant melanoma can occur in rare and nonsuspected locations, such as our patient’s tumour at the dentate line of the anal canal, which can manifest as a mucosal polyp mimicking the clinical presentation of other pathologies. This entity can develop in scleroderma patients with no clear relation, so close follow-up is advised. Radical surgery is the mainstay approach for anorectal melanoma despite the absence of a reliable treatment guideline. Immunotherapy is useful against anorectal melanoma. The concurrent incidence of scleroderma and anorectal melanoma make it challenging for an ideal approach as some therapies for one can aggravate the other.

## Ethical approval

The paper is only a case report and thus does not need any ethical approval.

## Patient consent

Written informed consent was obtained from the patient for the publication of this case report and accompanying images. A copy of the written consent is available for review by the Editor-in-Chief of this journal on request.

## Sources of funding

There were no sources of funding needed for this report.

## Author contribution

O.A. and R.M.: manuscript writing and literature research; H.O.: manuscript writing and collected the patient’s data; J.A.-D.: reviewed and revised the manuscript; L.A.-S: the mentor and patient’s dermatology care and follow-up; Z.A.: the guarantor, approved the final manuscript and performed the histopathological examination; S.K.: patient surgical care and follow-up.

## Conflicts of interest disclosure

The authors declare that they have no conflicts of interest.

## Guarantor

Zuhier Alshehabi, MD, FACP, PhD.

## Provenance and peer review

Not commissioned, externally peer-reviewed.
